# A Microbiological Indicator of Multidrug Resistance in Feline Urinary Tract Infections: Antimicrobial Resistance Patterns in Cats in Portugal

**DOI:** 10.3390/vetsci13050419

**Published:** 2026-04-24

**Authors:** Paula Segura Rodrigues, Bárbara Durão Feitor, Maria João Fonseca, André Marcelo Conceição Meneses, Joana Tavares de Oliveira

**Affiliations:** 1 Faculty of Veterinary Medicine, Lusófona University-Lisbon University Centre, 1749-024 Lisbon, Portugal; paulaseguramv@gmail.com (P.S.R.); p4795@ulusofona.pt (J.T.d.O.); 2 Hospital do Gato, 1400-146 Lisbon, Portugal; barbara.durao@hospitaldogato.com (B.D.F.); maria.fonseca@hospitaldogato.pt (M.J.F.); 3 I-MVET (Veterinary Medicine Research Group), Faculty of Veterinary Medicine, Lusófona University-Lisbon University Centre, 1749-024 Lisbon, Portugal; 4 Animal and Veterinary Research Center (CECAV), Lusófona University-Lisbon University Centre, 1749-024 Lisbon, Portugal

**Keywords:** feline urinary tract infection, antimicrobial resistance, multidrug resistance, urine culture, uropathogens, antimicrobial stewardship

## Abstract

Antimicrobial resistance (AMR) is an increasing concern in veterinary medicine and may compromise empirical treatment of feline urinary tract infections (UTIs). This retrospective observational study evaluated antimicrobial resistance patterns and their association with multidrug resistance (MDR) in bacterial isolates obtained from urine cultures of cats presented to a feline referral practice in Lisbon, Portugal, between January 2023 and December 2025. A total of 174 cats with positive urine cultures were included, yielding 178 bacterial isolates. *Escherichia coli* was the most frequent pathogen (57.30%), followed by *Enterococcus* spp. (16.98%) and *Staphylococcus* spp. (14.61%). Antimicrobial resistance was detected in 107/178 isolates (60.11%), and 76/178 (42.70%) were classified as multidrug-resistant. Extended-spectrum beta-lactamase-producing *Escherichia coli* and methicillin-resistant *Staphylococcus* strains were also identified. Resistance to penicillins and fluoroquinolones was significantly associated with MDR (*p* < 0.001). These findings highlight the high burden of antimicrobial resistance in feline urinary isolates in this clinical setting and support routine urine culture and susceptibility testing to guide therapeutic decision-making, particularly in recurrent, complicated, or high-risk cases.

## Simple Summary

Bacterial urinary tract infections in cats are often treated before culture results are available. However, this approach may fail when the infecting bacteria are resistant to commonly used antimicrobials. In this study, we evaluated bacteria isolated from feline urine cultures in a referral practice in Lisbon, Portugal, and found a high frequency of antimicrobial resistance, including multidrug-resistant isolates. *Escherichia coli* was the most common bacterium, followed by *Enterococcus* spp. and *Staphylococcus* spp. Resistance to penicillins and fluoroquinolones was significantly associated with multidrug resistance. These findings highlight the importance of urine culture and susceptibility testing to support more appropriate antimicrobial selection in cats with urinary tract infections.

## 1. Introduction

Urinary tract disease is frequently encountered in feline clinical practice, but bacterial urinary tract infection (UTI) represents only a subset of cats presenting with lower urinary tract signs. Non-infectious conditions, particularly idiopathic cystitis, are common; therefore, accurate differentiation between inflammatory and infectious disease is essential to guide antimicrobial therapy [1].

Despite this, antimicrobials are often prescribed empirically in cats with urinary signs. Empirical therapy without microbiological confirmation may promote the selection of resistant bacterial populations and compromise future therapeutic effectiveness [2]. Antimicrobial resistance has consequently become a major concern in veterinary medicine, with implications extending beyond animal health and affecting public health through the dissemination of resistant microorganisms.

Historically, bacterial UTIs in cats were considered uncommon, with early reports indicating low positive urine culture rates in the general feline population [2]. However, more recent investigations have demonstrated substantially higher prevalence, particularly in cats presenting with lower urinary tract signs and in selected feline populations with comorbidities such as chronic kidney disease, diabetes mellitus, and hyperthyroidism [1,3,4,5,6]. These findings suggest that bacterial infection may be underdiagnosed when microbiological testing is not performed.

Most feline UTIs are caused by bacteria originating from the host’s intestinal or urogenital microbiota. *Escherichia coli* is consistently reported as the primary uropathogen, while *Enterococcus* spp. and *Staphylococcus* spp. are among the most common Gram-positive isolates [4,7,8,9,10,11].

Increasing antimicrobial resistance has been documented among these pathogens, including multidrug-resistant isolates and clinically important resistance phenotypes [8,9,12,13,14,15]. In particular, *Enterococcus* spp. deserve special attention because they present intrinsic resistance to multiple antimicrobial classes, which may complicate therapeutic interpretation and treatment selection [16]. Similarly, *Staphylococcus* isolates are clinically relevant because methicillin resistance may substantially restrict therapeutic options [17].

Current clinical recommendations emphasize urine culture and antimicrobial susceptibility testing to guide treatment decisions [1,18]. However, antimicrobial therapy in clinical practice is still frequently initiated empirically, and knowledge of local resistance patterns is therefore essential for rational antimicrobial use. In Portugal, published data specifically addressing antimicrobial resistance in feline urinary isolates remain limited.

In addition to describing resistance prevalence, identifying microbiological features associated with multidrug resistance (MDR) may have practical clinical value. If specific susceptibility patterns are consistently associated with broader resistance, clinicians may recognize isolates with increased likelihood of MDR and adjust treatment strategies accordingly.

Therefore, the aim of this study was to characterize antimicrobial resistance patterns in bacteria isolated from feline urine cultures and to investigate whether resistance to selected antimicrobial classes was associated with multidrug resistance in a clinical population of cats from Lisbon, Portugal.

## 2. Materials and Methods

### 2.1. Study Design and Population

A retrospective observational clinical microbiology study was conducted using laboratory and clinical records from cats evaluated at a feline-exclusive referral practice in Lisbon, Portugal, between January 2023 and December 2025. The study included cats of both sexes and all ages whose urine cultures yielded bacterial growth. The unit of analysis was the bacterial isolate obtained from urine culture.

### 2.2. Case Selection

Medical records were reviewed, and all cats with positive urine cultures were considered for inclusion. Only urine samples obtained by cystocentesis were included, in order to minimize contamination and improve the diagnostic reliability of quantitative urine culture [1,19,20]. Cases without bacterial growth and cases with incomplete microbiological records were excluded.

A total of 174 cats fulfilled the inclusion criteria, yielding 178 bacterial isolates. Most infections were monomicrobial, although a small number of polymicrobial infections were identified. Because the study objective focused on microbiological resistance profiles, the bacterial isolate was used as the analytical unit. This approach should be interpreted with caution, since a small number of cats contributed more than one isolate.

### 2.3. Clinical Data Collection

For each included case, demographic and clinical variables were retrieved from the medical records. These included age, sex, breed, presence of comorbidities, and previous antimicrobial therapy. Comorbidities were recorded as present or absent based on the clinical diagnosis reported in the medical record, and specific chronic diseases were also tabulated during data extraction. Previous antimicrobial therapy was recorded from the clinical history when documented in the medical file. However, because the retrospective records did not consistently provide a precise time window for prior antimicrobial exposure, this variable should be interpreted with caution.

### 2.4. Urine Collection, Culture, and Bacterial Identification

Urine samples had been collected aseptically by cystocentesis during routine clinical care and submitted to the same external diagnostic laboratory for microbiological analysis. Cystocentesis was selected as the sampling method because it is considered the preferred technique for urine culture in cats, owing to its lower contamination risk when compared with voided or catheter-obtained samples [1,19,20].

Bacterial culture and isolate identification were performed by the external laboratory using routine microbiological procedures, including VITEK^®^ system (bioMérieux, Marcy-l’Étoile, France) identification. Isolates were identified to species or genus level according to the information available in the laboratory reports. In the final dataset, *Escherichia coli* was predominantly identified at species level, whereas some Gram-positive isolates, particularly *Enterococcus* spp. and *Staphylococcus* spp., were recorded at genus level.

### 2.5. Antimicrobial Susceptibility Testing

Antimicrobial susceptibility testing was performed using minimum inhibitory concentration (MIC)-based panels, following the routine diagnostic workflow of the laboratory. Two testing formats were represented in the dataset: a standard MIC panel and an extended MIC panel, as documented in the laboratory reports. The standard panel included ampicillin, amoxicillin–clavulanic acid, cefovecin, enrofloxacin, and gentamicin.

For isolates evaluated with the extended MIC panel, antimicrobials were grouped into the following classes for analysis: penicillins (ampicillin, amoxicillin, amoxicillin–clavulanic acid, penicillin, piperacillin), cephalosporins (cefovecin, cephalotin, cefoxitin, cefotaxime, ceftazidime), fluoroquinolones (enrofloxacin, ciprofloxacin, levofloxacin, pradofloxacin, marbofloxacin), aminoglycosides (gentamicin, amikacin, tobramycin), tetracyclines (tetracycline, doxycycline, minocycline), lincosamides (clindamycin), macrolides (erythromycin), sulfonamides and trimethoprim, phenicols (chloramphenicol), and nitrofurans (nitrofurantoin).

Interpretation of susceptibility results followed the laboratory criteria in use at the time of testing, based on the Clinical and Laboratory Standards Institute (CLSI; Wayne, PA, USA) [21]. Intermediate results (I) were considered non-susceptible in this analysis to provide a conservative estimate of antimicrobial resistance, consistent with common clinical microbiology practice.

### 2.6. Definition of Antimicrobial Resistance and Special Resistance Phenotypes

Isolates were classified as resistant or non-resistant according to the susceptibility testing results provided in the laboratory records. For the purposes of the present study, multidrug resistance (MDR) was defined as resistance to three or more antimicrobial classes, consistent with [22], and following the definition adopted in the original dataset and in this manuscript.

Special resistance phenotypes were also recorded when identified in the microbiological reports, including extended-spectrum beta-lactamase (ESBL)-producing *Escherichia coli* and methicillin-resistant *Staphylococcus* strains.

Because *Enterococcus* spp. may display intrinsic resistance to multiple antimicrobial classes, the interpretation of resistance patterns in this genus requires caution, particularly when discussing MDR frequencies and therapeutic implications [16,18].

### 2.7. Statistical Analysis

Descriptive statistics were used to summarize the study population, bacterial distribution, and antimicrobial resistance patterns. Inferential analyses were performed to assess: (i) the association between bacterial group and the presence of antimicrobial resistance; (ii) the association between host-related variables and multidrug resistance (MDR); and (iii) the association between resistance to selected antimicrobial classes and MDR.

Associations between categorical variables were evaluated using the chi-square test or Fisher’s exact test, as appropriate. Effect size for categorical associations was expressed using Cramer’s V. Odds ratios (ORs) with 95% confidence intervals (95% CIs) were calculated from 2 × 2 contingency tables to quantify the strength of association between selected variables and MDR. For host-related variables, ORs were calculated within the subgroup of resistant isolates (*n* = 107), comparing monoresistant and multidrug-resistant isolates. For antimicrobial class analyses, ORs were calculated using all isolates for which class-level resistance status could be classified. When a contingency table contained a zero cell, a continuity correction was applied for estimation of the OR and 95% CI.

Particular attention was given to resistance to penicillins and fluoroquinolones because these antimicrobial classes showed the strongest association with MDR in the dataset analyzed. Statistical significance was set at *p* < 0.05. Analyses were performed using JASP software (JASP Team, University of Amsterdam, Amsterdam, The Netherlands), version 0.95.4.

Given the retrospective design and the small number of cats contributing more than one isolate, results from isolate-level inferential analyses should be interpreted cautiously. In addition, no correction for multiple comparisons was applied in the original analytical framework, which should be considered when interpreting statistically significant associations.

### 2.8. Ethical Considerations

The study used retrospective clinical and laboratory data obtained during routine veterinary diagnostic procedures. No additional procedures were performed on animals for research purposes. Urine samples were collected as part of routine clinical investigation, and only anonymized clinical and microbiological information was analyzed.

## 3. Results

### 3.1. Population Characteristics

A total of 174 cats with positive urine cultures were included, yielding 178 bacterial isolates. Most cases corresponded to monomicrobial infection (170/174; 97.7%), whereas 4/174 cats (2.3%) had polymicrobial infections, accounting for the difference between the number of cats and the number of isolates analyzed. The study population consisted predominantly of females (117/174; 67.24%) and mixed-breed cats (151/174; 86.78%). The mean age was 11.4 years, and the highest frequency of infection was observed in cats aged 11–15 years (73/174; 41.95%) (Table 1). The age range of the study population was 1 to 20 years.

### 3.2. Bacterial Distribution

The distribution of bacterial isolates is summarized in Table 2. A total of 178 bacterial isolates were identified. *Escherichia coli* was the predominant uropathogen, representing 102/178 isolates (57.30%). *Enterococcus* spp. was the second most frequent group, with 30/178 isolates (16.98%), followed by *Staphylococcus* spp., with 26/178 isolates (14.61%). Other bacterial agents accounted for the remaining 20/178 isolates (11.24%).

### 3.3. Overall Antimicrobial Resistance

The overall antimicrobial resistance profile is presented in Table 3. Among the 178 isolates analyzed, 107/178 (60.11%) showed resistance to at least one antimicrobial, whereas 71/178 (39.89%) showed no documented antimicrobial resistance. When resistance categories were examined in more detail, 31/178 isolates (17.41%) were classified as resistant to a single antimicrobial, and 76/178 (42.70%) fulfilled the study definition of multidrug resistance (MDR).

Special resistance phenotypes were also identified, as shown in Table 3. Extended-spectrum beta-lactamase (ESBL)-producing isolates were detected in 6/178 isolates (3.37%), corresponding to 6/107 (5.61%) of resistant isolates. Methicillin-resistant *Staphylococcus* strains were identified in 3/178 isolates (1.69%), corresponding to 3/107 (2.80%) of resistant isolates.

A statistically significant association was observed between bacterial group and the presence of antimicrobial resistance (*p* < 0.001), with *Escherichia coli* and *Enterococcus* spp. showing the highest proportions of resistant isolates.

### 3.4. Resistance Patterns by Bacterial Group

The frequency of multidrug resistance within the main bacterial groups is shown in Table 4. Among *Escherichia coli* isolates, 44/102 (43.14%) were classified as multidrug-resistant, including ESBL-producing isolates. Among *Enterococcus* spp., 17/30 (56.67%) were multidrug-resistant. *Staphylococcus* spp. accounted for 26/178 isolates overall, and 3/26 (11.54%) were multidrug-resistant, including methicillin-resistant strains. These findings indicate that, although *Escherichia coli* was the most frequent pathogen overall, *Enterococcus* spp. showed the highest proportional representation of MDR within the major bacterial groups identified in the study.

### 3.5. Antimicrobials Involved in Monoresistance and Multidrug Resistance

The antimicrobials most frequently involved in monoresistance and multidrug resistance are summarized in Table 5. Among the 31 monoresistant isolates, ampicillin was the most frequently involved antimicrobial, accounting for 15/31 cases (48.39%), followed by cefovecin in 10/31 cases (32.26%).

Within the multidrug-resistant subgroup evaluated using the standard minimum inhibitory concentration (MIC) panel (*n* = 57), the highest resistance frequencies were observed for amoxicillin–clavulanic acid (47/57; 82.46%) and ampicillin (44/57; 77.19%).

Within the multidrug-resistant subgroup evaluated using the extended MIC panel (*n* = 19), universal resistance to penicillins was observed (19/19; 100%). High resistance frequencies were also identified for fluoroquinolones (18/19; 94.74%) and cephalosporins (17/19; 89.47%). In this subgroup, complex resistance profiles involving multiple antimicrobial classes were observed, with some isolates showing resistance to up to 13 different drugs.

### 3.6. Clinical Host-Related Variables and Multidrug Resistance

The distribution of clinical host-related variables and their association with multidrug resistance are presented in Table 6. Among the resistant subgroup (*n* = 107), 62/107 cats (57.9%) had at least one chronic comorbidity at the time of sample collection, whereas 45/107 (42.1%) had no documented chronic comorbidity. Chronic comorbidities were present in 16/31 (51.61%) monoresistant cases and in 46/76 (60.53%) multidrug-resistant cases.

As shown in Table 6, when specific chronic comorbidities were examined, chronic kidney disease was the most prevalent, affecting 53/107 resistant cases (49.5%), followed by other chronic comorbidities (36/107; 33.6%) and hyperthyroidism (9/107; 8.4%). Diabetes mellitus was uncommon, being recorded in only 1/107 resistant cases (0.9%). Cats with multiple comorbidities were counted in each applicable disease category; therefore, the sum of specific comorbidities exceeded the total number of cats with any chronic comorbidity.

As summarized in Table 6, no significant association was found between host-related variables and multidrug resistance. The presence of any chronic comorbidity was not associated with MDR (OR 1.44, 95% CI 0.62–3.33; *p* = 0.397). Likewise, no significant associations were identified for chronic kidney disease (OR 1.21, 95% CI 0.53–2.81; *p* = 0.650), diabetes mellitus (OR 0.20, 95% CI 0.01–6.04; *p* = 0.116), hyperthyroidism (OR 1.24, 95% CI 0.24–6.52; *p* = 0.797), other comorbidities (OR 1.33, 95% CI 0.52–3.39; *p* = 0.585), previous antimicrobial therapy (OR 0.82, 95% CI 0.35–1.93; *p* = 0.654), or clinical signs of lower urinary tract disease (OR 2.48, 95% CI 0.81–7.57; *p* = 0.103).

Overall, host-related factors did not show a statistically significant association with multidrug resistance in the analytical dataset used in this study.

### 3.7. Association Between Resistance to Antimicrobial Classes and Multidrug Resistance

The association between resistance to selected antimicrobial classes and multidrug resistance is presented in Table 7. In contrast to host-related variables, resistance to selected antimicrobial classes showed a statistically significant association with multidrug resistance.

As shown in Table 7, resistance to penicillins was significantly associated with MDR (OR 35.48, 95% CI 15.12–83.22; *p* < 0.001). Similarly, resistance to fluoroquinolones was significantly associated with MDR (OR 24.50, 95% CI 8.19–73.33; *p* < 0.001).

Taken together, as summarized in Table 7, these findings indicate that resistance to penicillins and fluoroquinolones was closely associated with broader resistance patterns in the present dataset, whereas host-related variables were not.

## 4. Discussion

The present study identified a high frequency of antimicrobial resistance among bacterial isolates recovered from feline urine cultures, with 60.11% of isolates showing resistance to at least one antimicrobial and 42.70% fulfilling the study definition of multidrug resistance. These findings reinforce that the clinical challenge in feline urinary tract infections is not limited to confirming bacterial infection but also includes selecting an effective antimicrobial in a context in which empirical therapy may be unreliable. This has clear implications for antimicrobial stewardship in companion animal practice, particularly because antimicrobial treatment is still frequently initiated before culture and susceptibility results are available [1,13,23].

The bacterial distribution observed in this study is consistent with previous reports describing *Escherichia coli* as the predominant feline uropathogen, followed by Gram-positive organisms such as *Enterococcus* spp. and *Staphylococcus* spp. [4,7,9,10,11]. In the present population, *E. coli* accounted for 57.30% of isolates, followed by *Enterococcus* spp. (16.98%) and *Staphylococcus* spp. (14.61%). This overall distribution supports the interpretation that the study population is representative of feline urinary infections encountered in referral practice rather than microbiologically atypical.

From a resistance standpoint, the proportion of multidrug-resistant isolates found in this study was substantial. This prevalence appears clinically relevant when compared with the broader literature from companion animal urinary isolates in Europe and elsewhere, where increasing resistance and the emergence of multidrug-resistant pathogens have been repeatedly documented [8,9,12,15,24]. Although direct comparison between studies must be made cautiously because of differences in case selection, bacterial composition, susceptibility panels, and interpretive criteria, the high MDR frequency observed here supports the view that antimicrobial resistance in feline urinary isolates is an important and current clinical problem in Portugal.

The predominance of *E. coli* among isolates is expected and agrees with its established role as the principal uropathogen in feline urinary infections [4,7,11]; However, the proportional frequency of multidrug resistance was particularly notable in *Enterococcus* spp., in which 56.67% of isolates were classified as MDR. This finding should be interpreted carefully. *Enterococcus* species are recognized opportunistic pathogens in urinary tract infections, especially in animals with concurrent disease, prior antimicrobial exposure, or repeated medical intervention, but they also possess intrinsic resistance to multiple antimicrobial classes [16,18]. Therefore, their apparent MDR burden may partly reflect intrinsic microbiological characteristics in addition to acquired resistance mechanisms. For this reason, the clinical role of *Enterococcus* spp. in feline urine cultures should not be oversimplified as either contaminant or primary pathogen; rather, interpretation should be individualized according to sampling method, quantitative growth, urinalysis findings, and clinical context.

The detection of ESBL-producing *Escherichia coli* and methicillin-resistant *Staphylococcus* isolates further emphasizes the clinical relevance of the resistance patterns identified in this study. These phenotypes are of particular concern because they may severely restrict therapeutic options and increase the likelihood of empirical treatment failure. Their presence also aligns with previous reports highlighting the emergence and dissemination of clinically important resistance mechanisms in companion animal bacterial isolates [12,13,14]. Even though the absolute number of such isolates in the present study was limited, their identification in routine feline urinary infections reinforces the need for cautious antimicrobial selection and culture-based decision-making.

One of the most clinically relevant findings of this study was the statistically significant association between resistance to penicillins and fluoroquinolones and the occurrence of multidrug resistance. These classes are particularly important in practice because they are commonly represented in empirical treatment strategies for urinary disease in small animals. The present findings suggest that resistance to either class may function as a practical microbiological warning sign of broader resistance. However, this result should be interpreted as an association rather than as definitive proof of predictive performance, because the current analytical approach did not include multivariable modelling or diagnostic performance measures such as sensitivity and specificity. Nonetheless, the calculated odds ratios with 95% confidence intervals support the interpretation that isolates resistant to penicillins or fluoroquinolones were substantially more likely to be multidrug-resistant. Accordingly, the data supports cautious clinical interpretation of penicillin and fluoroquinolone resistance as markers associated with MDR, rather than as validated stand-alone predictors.

In practical terms, this association remains relevant. When resistance to penicillins or fluoroquinolones is identified in feline urinary isolates, clinicians should be aware that broader resistance may also be present and that repeated empirical antimicrobial changes may not be an appropriate strategy. Instead, these findings support prioritizing definitive culture-guided treatment and limiting unnecessary antimicrobial exposure. This interpretation is consistent with current antimicrobial stewardship principles and with recommendations favoring urine culture and susceptibility testing whenever possible in complicated, recurrent, or higher-risk feline urinary tract infections [1,18,23].

In contrast, host-related factors such as age, sex, comorbidities, and previous antimicrobial therapy were not significantly associated with multidrug resistance in the present dataset. This finding should not be interpreted to mean that host characteristics are irrelevant in feline urinary disease. On the contrary, previous studies have shown that older cats and cats with chronic kidney disease, diabetes mellitus, and hyperthyroidism may be at increased risk of bacterial urinary tract infection or bacteriuria [1,3,4,5,25]. Rather, the present results suggest that factors predisposing to infection may not necessarily coincide with factors associated with broader resistance patterns in this retrospective dataset. Methodological limitations must also be considered, including the retrospective nature of data collection, incomplete standardization of prior clinical history, the lack of a precise time window for previous antimicrobial exposure, and the relatively limited statistical granularity available for host-level analyses.

The absence of significant associations between host variables and MDR may also reflect the design of the study rather than a true lack of biological effect. The sample was derived from a feline-exclusive referral practice, which may have enriched the population for more complex, recurrent, previously treated, or comorbid cases. In addition, a small number of cats contributed more than one isolate, and inferential analysis was performed at isolate level. These factors may have affected the independence of observations and reduced the capacity to detect subtle host-level relationships. Therefore, the present findings should be interpreted cautiously and should not be taken as conclusive evidence that clinical history is unrelated to resistance risk.

This study has several limitations that should be acknowledged. First, it was retrospective and based on data from a single feline referral center, which limits generalizability and introduces potential referral and selection bias. Second, although all samples were collected by cystocentesis, which strengthens microbiological reliability, some key clinical variables were not uniformly documented in the medical records, particularly the precise timing and nature of previous antimicrobial therapy. Third, bacterial identification was not uniformly resolved to species level for all Gram-positive isolates, especially within *Staphylococcus* spp., which limits more refined epidemiological and clinical interpretation. Fourth, multidrug resistance was assessed using susceptibility panels generated during routine laboratory practice, and although interpretation followed CLSI-based laboratory criteria, not all isolates were necessarily evaluated with identical antimicrobial panel breadth. Fifth, no correction for multiple comparisons was applied in the statistical analysis, and this should be considered when interpreting significant associations. Finally, molecular characterization of resistance mechanisms was not performed, preventing distinction between phenotypic resistance patterns and the underlying genetic determinants.

Despite these limitations, the study has important strengths. It evaluated a relatively large clinical feline population with culture-confirmed infection, used cystocentesis-derived urine samples, and identified both general resistance burden and specific microbiological patterns associated with multidrug resistance. Taken together, the findings provide clinically relevant local data from Portugal and reinforce the importance of microbiological diagnosis in feline urinary tract infections.

Overall, the present results indicate that antimicrobial resistance is common in feline urinary isolates in this setting and that multidrug resistance is not rare. The high prevalence of resistant and multidrug-resistant isolates, together with the detection of ESBL-producing *E. coli* and methicillin-resistant *Staphylococcus* strains, highlights the limitations of empirical therapy. Resistance to penicillins and fluoroquinolones was significantly associated with MDR and may therefore alert clinicians to the possibility of broader resistance, although further studies using more robust predictive models are needed before this association can be interpreted as a validated predictive tool.

## 5. Conclusions

This study demonstrated a high frequency of antimicrobial resistance among bacteria isolated from feline urine cultures in a referral clinical setting in Lisbon, Portugal. *Escherichia coli* was the predominant uropathogen, followed by *Enterococcus* spp. and *Staphylococcus* spp., and a substantial proportion of isolates fulfilled the study definition of multidrug resistance.

The identification of ESBL-producing *Escherichia coli* and methicillin-resistant *Staphylococcus* strains further highlights the clinical relevance of these findings and reinforces the limitations of empirical antimicrobial therapy in feline urinary tract infections. In this dataset, resistance to penicillins and fluoroquinolones was significantly associated with multidrug resistance, suggesting that these resistance patterns may alert clinicians to the possibility of broader antimicrobial resistance.

Taken together, the results support the routine use of urine culture and antimicrobial susceptibility testing in feline urinary tract infections, particularly in recurrent, complicated, or high-risk cases. Further studies using broader datasets and more robust analytical models are warranted to better define microbiological markers associated with multidrug resistance in feline urinary isolates.

## Figures and Tables

**Table 1:** Population characteristics of cats with positive urine cultures.

Variable	Number (*n*)	Percentage (%)
Infection type		
Monomicrobial	170	97.7
Polymicrobial	4	2.3
Sex		
Female	117	67.24
Male	57	32.76
Breed		
Mixed breed	151	86.78
Purebred	23	13.22
Age		
≤2 years	9	5.17
3–5 years	20	11.49
6–10 years	33	18.97
11–15 years	73	41.95
>15 years	39	22.41

**Table 2:** Distribution of bacterial isolates obtained from feline urine cultures (*n* = 178).

Bacterial Isolate	Number (*n*)	Percentage (%)
*Escherichia coli*	102	57.30
*Enterococcus* spp.	30	16.98
*Staphylococcus* spp.	26	14.61
Other bacteria	20	11.24

**Table 3:** Overall antimicrobial resistance, multidrug resistance, and special resistance phenotypes among bacterial isolates from feline urine cultures (*n* = 178).

Category	Number of Isolates (*n*)	Percentage of Total Isolates (%)
Any antimicrobial resistance	107	60.11
Multidrug resistance (MDR)	76	42.7
ESBL-producing isolates	6	3.37
Methicillin-resistant *Staphylococcus* spp. (MRS)	3	1.69

Note: ESBL corresponded to 5.61% of resistant isolates (6/107), and MRS corresponded to 2.80% of resistant isolates (3/107).

**Table 4:** Frequency of multidrug resistance within the main bacterial groups.

Bacterial Group	Total (*n*)	MDR (*n*)	MDR (%)
*Escherichia coli*	102	44	43.14
*Enterococcus* spp.	30	17	56.67
*Staphylococcus* spp.	26	3	11.54

**Table 5:** Antimicrobials most frequently involved in monoresistance and MDR.

Antimicrobial	Monoresistance (*n* = 31)	MDR-Standard MIC (*n* = 57)	MDR-Extended MIC (*n* = 19)
Penicillins Ampicillin	- 15 (48.39%)	- 44 (77.19%)	19 (100%) 18 (94.74%)
Amoxicillin–clavulanic acid	-	47 (82.46%)	19 (100%)
Cephalosporins Cefovecin	10 (32.26%)	-	17 (89.47%) 16 (84.21%)
Fluoroquinolones Enrofloxacin	-	-	18 (94.74%) 18 (94.74%)
Ciprofloxacin	-	-	11 (57.89%)
Levofloxacin	-	-	9 (47.37%)

**Table 6:** Clinical host-related variables and their association with multidrug resistance.

Variable	Monoresistant (*n* = 31)	MDR (*n* = 76)	Total (*n* = 107)	OR (95% CI)	*p*-Value	Effect Size (Cramer’s V)
Chronic comorbidity					0.397	0.082
Present	16 (51.61%)	46 (60.53%)	62 (57.94%)	1.44 (0.62–3.33)		
Absent	15 (48.39%)	30 (39.47%)	45 (42.06%)	Reference		
Chronic kidney disease					0.650	0.044
Present	14 (45.16%)	39 (51.32%)	53 (49.53%)	1.21 (0.53–2.81)		
Absent	17 (54.84%)	37 (48.68%)	54 (50.47%)	Reference		
Diabetes mellitus *					0.116	0.152
Present	1 (3.23%)	0 (0.00%)	1 (0.93%)	0.20 (0.01–6.04)		
Absent	30 (96.77%)	76 (100.00%)	106 (99.07%)	Reference		
Hyperthyroidism					0.797	0.025
Present	2 (6.45%)	7 (9.21%)	9 (8.41%)	1.24 (0.24–6.52)		
Absent	29 (93.55%)	69 (90.79%)	98 (91.59%)	Reference		
Other comorbidities					0.585	0.281
Present	9 (29.03%)	27 (35.53%)	36 (33.64%)	1.33 (0.52–3.39)		
Absent	22 (70.97%)	49 (64.47%)	71 (66.36%)	Reference		
Previous antimicrobial therapy					0.654	0.043
Yes	18 (58.06%)	42 (55.26%)	60 (56.07%)	0.82 (0.35–1.93)		
No	13 (41.94%)	34 (44.74%)	47 (43.93%)	Reference		
Clinical signs of LUTD					0.103	0.158
Present	25 (80.65%)	69 (90.79%)	94 (87.85%)	2.48 (0.81–7.57)		
Absent	6 (19.35%)	7 (9.21%)	13 (12.15%)	Reference		

* For diabetes mellitus, the odds ratio and 95% confidence interval were estimated using a continuity correction because one cell contained zero observations. Cats with multiple comorbidities were included in more than one comorbidity category.

**Table 7:** Association between resistance to selected antimicrobial classes and multidrug resistance.

Antimicrobial Class	MDR Among Resistant Isolates *n*/N (%)	MDR Among Susceptible Isolates *n*/N (%)	OR (95% CI)	*p*-Value
Penicillins	80.49%	10.42%	35.48 (15.12–83.22)	<0.001
Fluoroquinolones	90.48%	27.94%	24.50 (8.19–73.33)	<0.001

## Data Availability

The data presented in this study are available from the corresponding author upon reasonable request. The data are not publicly available due to privacy and clinical record confidentiality.

## References

[1] Dorsch R., Teichmann-Knorrn S., Sjetne Lund H. (2019). Urinary Tract Infection and Subclinical Bacteriuria in Cats: A Clinical Update. J. Feline Med. Surg..

[2] Bartges J.W., Barsanti J.A. (2000). Bacterial Urinary Tract Infections in Cats. Bact. Urin. Tract Infect. Cats.

[3] Mayer-Roenne B., Goldstein R.E., Erb H.N. (2007). Urinary Tract Infections in Cats with Hyperthyroidism, Diabetes Mellitus and Chronic Kidney Disease. J. Feline Med. Surg..

[4] Martinez-Ruzafa I., Kruger J.M., Miller R., Swenson C.L., Bolin C.A., Kaneene J.B. (2012). Clinical Features and Risk Factors for Development of Urinary Tract Infections in Cats. J. Feline Med. Surg..

[5] White J.D., Cave N.J., Grinberg A., Thomas D.G., Heuer C. (2016). Subclinical Bacteriuria in Older Cats and Its Association with Survival. J. Vet. Intern. Med..

[6] Puchot M.L., Cook A.K., Pohlit C. (2017). Subclinical Bacteriuria in Cats: Prevalence, Findings on Contemporaneous Urinalyses and Clinical Risk Factors. J. Feline Med. Surg..

[7] Litster A., Thompson M., Moss S., Trott D. (2011). Feline Bacterial Urinary Tract Infections: An Update on an Evolving Clinical Problem. Vet. J..

[8] Marques C., Gama L.T., Belas A., Bergström K., Beurlet S., Briend-Marchal A., Broens E.M., Costa M., Criel D., Damborg P. (2016). European Multicenter Study on Antimicrobial Resistance in Bacteria Isolated from Companion Animal Urinary Tract Infections. BMC Vet. Res..

[9] Rampacci E., Bottinelli M., Stefanetti V., Hyatt D.R., Sgariglia E., Coletti M., Passamonti F. (2018). Antimicrobial Susceptibility Survey on Bacterial Agents of Canine and Feline Urinary Tract Infections: Weight of the Empirical Treatment. J. Glob. Antimicrob. Resist..

[10] Mazda D., Lau S.F., Omar S. (2023). Clinical Investigation of Feline Lower Urinary Tract Disease, Pathogenic Bacteria and Their Antibiotic Sensitivity at University Veterinary Hospital, Universiti Putra Malaysia. Thai J. Vet. Med..

[11] Yudhanto S., Maddox C.W., Varga C., Hung C.-C. (2025). Prevalence and Antimicrobial Resistance of Bacteria Isolated from Urine Samples of Cats with Urinary Tract Infections in Illinois, United States of America. Res. Vet. Sci..

[12] Marques C., Belas A., Franco A., Aboim C., Gama L.T., Pomba C. (2018). Increase in Antimicrobial Resistance and Emergence of Major International High-Risk Clonal Lineages in Dogs and Cats with Urinary Tract Infection: 16 Year Retrospective Study. J. Antimicrob. Chemother..

[13] Yudhanto S., Hung C.-C., Maddox C.W., Varga C. (2022). Antimicrobial Resistance in Bacteria Isolated From Canine Urine Samples Submitted to a Veterinary Diagnostic Laboratory, Illinois, United States. Front. Vet. Sci..

[14] Menezes J., Frosini S.-M., Weese S., Perreten V., Schwarz S., Amaral A.J., Loeffler A., Pomba C. (2024). Transmission Dynamics of ESBL/AmpC and Carbapenemase-Producing Enterobacterales between Companion Animals and Humans. Front. Microbiol..

[15] Wei A.Y., Thompson M.F., Worthing K.A., Venturini C., Brookes V.J., Norris J.M. (2025). Frequency and Antimicrobial Susceptibility Profiles of Bacterial Species Isolated from Canine and Feline Urine Samples in Sydney, Australia, 2012–2021. Vet. Microbiol..

[16] Hollenbeck B.L., Rice L.B. (2012). Intrinsic and Acquired Resistance Mechanisms in Enterococci. Intrinsic Acquir. Resist. Mech. Enterococci.

[17] Peacock S.J., Paterson G.K. (2015). Mechanisms of Methicillin Resistance in *Staphylococcus aureus*. Annu. Rev. Biochem..

[18] Papich M.G. (2016). Antimicrobial Susceptibility Testing for Feline Urinary Tract Isolates. J. Feline Med. Surg..

[19] Pereira A., Jota Baptista C., Oliveira P.A., Coelho A.C. (2024). Urinary Tract Bacterial Infections in Small Animal Practice: Clinical and Epidemiological Aspects. Vet. Stn. (Online).

[20] Taylor S., Boysen S., Buffington T., Chalhoub S., Defauw P., Delgado M.M., Gunn-Moore D., Korman R. (2025). 2025 iCatCare Consensus Guidelines on the Diagnosis and Management of Lower Urinary Tract Diseases in Cats. J. Feline Med. Surg..

[21] Clinical and Laboratory Standards Institute (2024). Performance Standards for Antimicrobial Disk and Dilution Susceptibility Tests for Bacteria Isolated from Animals.

[22] Magiorakos A.-P., Srinivasan A., Carey R.B., Carmeli Y., Falagas M.E., Giske C.G., Harbarth S., Hindler J.F., Kahlmeter G., Olsson-Liljequist B. (2012). Multidrug-Resistant, Extensively Drug-Resistant and Pandrug-Resistant Bacteria: An International Expert Proposal for Interim Standard Definitions for Acquired Resistance. Clin. Microbiol. Infect..

[23] Weese J.S., Stull J.W., Evason M., Webb J., Ballance D., McKee T., Bergman P.J. (2022). A Multicenter Study of Antimicrobial Prescriptions for Cats Diagnosed with Bacterial Urinary Tract Disease. J. Feline Med. Surg..

[24] Darwich L., Seminati C., Burballa A., Nieto A., Durán I., Tarradas N., Molina-López R.A. (2021). Antimicrobial Susceptibility of Bacterial Isolates from Urinary Tract Infections in Companion Animals in Spain. Vet. Rec..

[25] White J.D., Stevenson M., Malik R., Snow D., Norris J.M. (2013). Urinary Tract Infections in Cats with Chronic Kidney Disease. J. Feline Med. Surg..

